# Enhancing Survivorship Care Planning for Patients With Localized Prostate Cancer Using a Couple-Focused mHealth Symptom Self-Management Program: Protocol for a Feasibility Study

**DOI:** 10.2196/resprot.9118

**Published:** 2018-02-26

**Authors:** Lixin Song, Kaitlyn L Dunlap, Xianming Tan, Ronald C Chen, Matthew E Nielsen, Rebecca L Rabenberg, Josephine K Asafu-Adjei, Bridget F Koontz, Sarah A Birken, Laurel L Northouse, Deborah K Mayer

**Affiliations:** ^1^ School of Nursing University of North Carolina Chapel Hill, NC United States; ^2^ Lineberger Comprehensive Cancer Center School of Medicine University of North Carolina Chapel Hill, NC United States; ^3^ University of North Carolina Chapel Hill, NC United States; ^4^ School of Medicine Duke University Durham, NC United States; ^5^ School of Nursing University of Michigan Ann Arbor, MI United States

**Keywords:** survivorship, prostate cancer, symptom, randomized trial, mHealth, caregiver, Patient Reported Outcome Measures

## Abstract

**Background:**

This project explores a new model of care that enhances survivorship care planning and promotes health for men with localized prostate cancer transitioning to posttreatment self-management. Survivorship care planning is important for patients with prostate cancer because of its high incidence rate in the United States, the frequent occurrence of treatment-related side effects, and reduced quality of life (QOL) for both men and their partners. A key component of comprehensive survivorship care planning is survivorship care plans (SCPs), documents that summarize cancer diagnosis, treatment, and plans for follow-up care. However, research concerning the effectiveness of SCPs on patient outcomes or health service use has thus far been inconclusive. SCPs that are tailored to individual patients’ needs for information and care may improve effectiveness.

**Objective:**

This study aims to examine the feasibility of an enhanced survivorship care plan (ESCP) that integrates a symptom self-management mHealth program called Prostate Cancer Education and Resources for Couples (PERC) into the existing standardized SCP. The specific aims are to (1) examine the feasibility of delivering ESCPs and (2) to estimate the magnitude of benefit of ESCPs.

**Methods:**

We will use a two-group randomized controlled pretest-posttest design and collect data at baseline (T1) and 4 months later (T2) among 50 patients completing initial treatment for localized prostate cancer and their partners. First, we will assess the feasibility of ESCP by recruitment, enrollment, and retention rates; program satisfaction with the ESCP; and perceived ease of use of the ESCP. To achieve the secondary aim, we will compare the ESCP users with the standardized SCP users and assess their primary outcomes of QOL (overall, physical, emotional, and social QOL); secondary outcomes (reduction in negative appraisals and improvement in self-efficacy, social support, and health behaviors to manage symptoms); and number of visits to posttreatment care services between T1 and T2. We will assess the primary and secondary outcomes using measurements with sound psychometrical properties. We will use a qualitative and quantitative mixed methods approach to achieve the research aims.

**Results:**

This project is ongoing and will be completed by the end of 2018.

**Conclusions:**

The results from this study will help design a definitive randomized trial to test the efficacy of the ESCPs, a potentially scalable program, to enhance supportive care for prostate cancer patients and their families.

## Introduction

A key component of cancer survivorship care planning is survivorship care plans (SCPs), documents intended in part to improve survivors’ understanding of treatment-related symptoms, and ultimately, to improve patient outcomes such as quality of life (QOL) by summarizing cancer diagnosis, treatment, and follow-up care [1-3]. SCP use is recommended by several high-profile organizations such as the Commission on Cancer and Institute of Medicine (IOM) [1-4]. To date, research on SCPs has been largely empirical and inconclusive regarding whether the use of standardized SCPs improves patient outcomes [5-8]. The limited research demonstrating SCP efficacy may relate in part to the failure of standardized SCPs to be tailored to patient-specific information and care needs during care transition [9-11]. To enhance survivorship care planning, SCPs, as part of routine care, may create a channel for distributing interventions to patients to improve their symptom self-management and other outcomes [12,13].

### Gaps in Cancer Survivorship Care Planning

Survivorship care planning for patients with prostate cancer is particularly important because of the high incidence rates of prostate cancer among men in the United States [14], the frequent occurrence of side effects due to treatments with curative intent [15-22] (eg, urinary, sexual, bowel, and hormonal symptoms; emotional distress; pain; fatigue; and sleep disturbance), and reduced QOL caused by these symptoms. Most patients are reluctant to talk with professionals or at support groups about their prostate cancer and its impact on their lives due to the sensitive nature of prostate cancer and its symptoms [23]. For patients in an intimate relationship, the effects of prostate cancer symptoms on their partners’ QOL are similar to or worse than the effects on their own QOL [24,25]. Management of these negative effects has been an unaddressed supportive care need for survivors and their partners [26-28]. The IOM [3] and American Cancer Society (ACS) [29] cancer care guidelines call for programs that address treatment-related effects, promote healthy behaviors, and maintain QOL for patients and their families.

### Using mHealth to Enhance Survivorship Care Planning

To address the unmet care needs of patients and their partners, our interdisciplinary research team developed a couple-focused tailored prostate cancer education mHealth program, Prostate Cancer Education and Resources for Couples (PERC) [30] based on scientific evidence and input from three groups of stakeholders: patients, partners, and cancer care providers. The theory-driven PERC program aims to improve QOL for both patients and partners through tailored content and a set of features that provide information and skills training, as well as increase their self-efficacy, social support, and health behaviors for symptom management [30]. In the two pilot feasibility studies we conducted during PERC development, prostate cancer patients and partners reported high satisfaction with PERC. They reported that PERC was simple and easy to use and that it provided quality information that improved their symptom management and QOL. Our pilot participants also suggested vigorously advertising PERC and “having the program available at physicians’ offices” so that “all prostate cancer patients and their families can access it.” [30].

To strengthen survivorship care planning for patients with localized prostate cancer and respond to our pilot participants’ suggestions, we proposed to use SCPs as a vehicle for consistent and timely delivery of PERC and to enhance the standardized SCPs (hereafter, ESCP: enhanced survivorship care plan). We used the theoretical framework adapted from the Transactional Model of Stress and Coping [31] and family systems theory [32-34] to guide the development of the ESCP for prostate cancer patients in an intimate relationship. In this framework, personal, couple, and cancer-related factors are precursors *(antecedents)* of patients’ and partners’ QOL (*primary outcomes*) and also have indirect effects on QOL through *secondary outcomes* including appraisals of symptoms and coping resources (self-efficacy in symptom management, social support, and healthy lifestyle behaviors). The framework shows that patients and partners manage prostate cancer symptoms interdependently (when one person functions poorly, the other person is negatively affected [33,35]). This shapes their appraisals, coping resources, and ultimately affects the QOL outcome of each of the individuals.

### Research Aims

In this proof-of-concept randomized controlled pilot trial, our primary objective is to examine the feasibility of delivering an ESCP (ie, standard SCP enhanced by the PERC program) to patients and partners. Our secondary objective of this study is to estimate the magnitude of potential benefit of the ESCPs. Compared with patients and partners who received the standardized SCP, we hypothesize that patients and partners using the ESCP will report greater improvement, from baseline (T1) to follow-up (T2), in QOL, self-efficacy in symptom management, social support, health behaviors to manage symptoms, and appraisals of prostate cancer symptoms. In addition, we hypothesize that patients receiving the standardized SCP and ESCP will differ in the number of visits to posttreatment care services between T1 and T2.

## Methods

### Study Design

This study will test the feasibility of a two-group randomized controlled pilot trial using prepost mixed-method design. Patients and their partners will be randomly assigned to the SCP (control) or the ESCP (intervention) groups. Couples will complete study measures at T1 (before randomization) and T2 (4 months later).

### Study Participants and Setting

A total of 50 men who have recently completed initial treatment for localized prostate cancer and their partners will participate in this feasibility and proof-of-concept study. The eligible patients must (1) be within 16 weeks of completing their initial treatment with curative intent for localized prostate cancer (ie, prostatectomy or radiotherapy with or without hormonal treatment) [36] at the genitourinary and radiation oncology clinics of the two comprehensive cancer centers in the southeast of United States; (2) not be receiving treatment currently or within the past year for another cancer; and (3) have a partner who is 18 years of age or older, not receiving cancer treatment currently or within the past year, and willing to participate.

Patients and their partners will be removed from this study if she or he is diagnosed with a new type of cancer, starts a new treatment for another cancer during the study period, or decides to withdraw from the study voluntarily. Patients and their partners will be excluded from the study if either partner does not read and speak English as evidenced by their understanding and responses to screening questions and self-reported ability to read English or has cognitive impairment (assessed by the short portable mental status questionnaire).

### Study Procedure

The research staff will use convenience sampling to recruit patients and partners from the genitourinary and radiation oncology clinics of two large comprehensive cancer centers in the southeast United States, where at least 400 men with localized prostate cancer receive treatment annually, and about 25% are African Americans, ensuring successful recruitment for this study. We will recruit couples based on procedures used successfully in the past by other researchers [37] and in our pilot study [30]. After Institutional Review Board approval, the project coordinator will identify potentially eligible patients using patient scheduling systems. The project coordinator then will see patients who meet the inclusion criteria before their SCP follow-up visit. The coordinator will provide study information, screen the patient and his partner for their eligibility and willingness to participate, obtain informed consent, and collect baseline data via telephone survey. For patients whose partners are not present at the clinic, the project coordinator will screen and consent the patients and partners and answer their questions via telephone after eligible patients give permission to contact their partners.

#### Randomization

After the T1 survey completion, couples will be randomized to the standardized SCP or the ESCP groups using a 1:1 ratio; 25 couples in each group (N=50 couples). The study statistician will generate, using an SAS program (SAS Institute Inc., Cary, NC, USA), a stratified *permuted block randomization plan with varying block sizes.* The randomization will be stratified by type of treatment (surgery, radiation, radiation plus hormonal therapy) because we believe that treatment type correlates with symptoms and QOL [38]. The health educator will administer this allocation sequence and send couples a letter and message via mail, email, and phone explaining their group assignment and study activities. After randomization, all participants will receive the standardized SCPs plus the Web link to our study website that is inserted at the end of the SCP using Smart Phrase in the electronic medical record Epic system. Following SCP delivery by clinicians, the interventionist will assign all participants their usernames and passwords and invite them to log into the study website embedded in the SCP via email, telephone, or mail. Other team members will be blinded to the treatment allocation until the end of the study, whereas the interventionist who knows the treatment allocation will not conduct surveys or interviews.

#### Control Condition

After logging into the study website that is embedded in the SCPs, control participants will be directed to the National Cancer Institute (NCI) prostate cancer website. A range of sources including NCI and ACS are routinely available in standardized SCPs to all patients. We utilize the auto-direction to the NCI website as an attention control to improve blindness during the randomization process. Furthermore, we include the NCI website to ensure that participants in the control group have structured access to evidence-based and guideline-adherent information and equivalence between control and experimental groups.

The use of SCPs is part of routine care at the genitourinary and radiation oncology clinics. After completing initial treatment for prostate cancer, patients’ clinicians will complete and print a standardized SCP adapted from the American Society of Clinical Oncology template, review it with the patient (and their families) in a private room behind a closed door or via telephone, and provide him with a copy. The SCP will also be sent to the patient’s primary care provider. The standardized SCP’s section about possible late- and long-term treatment effects provides a generic summary of the side effects of the patient’s treatment; options for managing the side effects; and recommendations for diet, physical activities, smoking cessation, and stress. All of this information is brief and is nonspecific to individual patients. There is no content about caregiver and caregiving issues during posttreatment survivorship in the standardized SCP.

#### Intervention

Participants randomized to the intervention group will receive the same SCP but will be directed to the PERC intervention website after logging into our study website instead of the control group’s NCI website. PERC includes 12 modules about how couples can work effectively as a team, assess and better manage prostate cancer treatment-related side effects and symptoms (including urinary and bowel problems, sexual dysfunction, hormonal symptoms, pain, fatigue, sleep disturbance, and stress), and improve healthy behaviors. PERC also facilitates social support for the patient and his partner via postmodule assignments, a moderated online forum, meetings with a health educator, and a resource center that connects participants and their partners to tools for symptom tracking and monitoring, as well as local and national support groups and resources. Participants in the intervention group will have 15 weeks to complete the PERC intervention.

#### Measurement and Data Collection

To achieve our primary objective of testing the feasibility of the ESCP, we will collect both quantitative and qualitative data.

**Table 1:** Summary of measures at baseline (T1) and 4 months post baseline (T2).

Variables and Measurement^a^			Data source^b^	Group	T1	T2	Cronbach alpha	PERC program^c^
**Aim 1: Feasibility of the ESCP**								
	Screening, enrollment, and retention rates: research activity logs; field notes		AD	Both^d^	Yes	Yes	N/A^e^	N/A
	Self-reported program use		PT, SP	Both		Yes	N/A	N/A
	Web activity: use NCI versus PERC or not, number of logins		Tracking	Both	Yes	Yes	N/A	Built in tracking system
	Program satisfaction and perceived ease of use: Usability Scale [39,40]		PT, SP	Both	Yes	Yes	N/A	N/A
	Participants’ experiences: exit interview		PT, SP, AD	Both	No	Yes	N/A	N/A
**Aim 2: Magnitude of benefits of the ESCPs (compared with couples using SCPs)**								
	**Primary outcomes**							
		Quality of life (overall, physical, emotional, and social well-being): functional assessment of chronic illness therapy general scale (27-item) [41,42]	PT, SP	Both	Yes	Yes	.90 [25,42]	All^f^
		Number of visits to post treatment care services: medical records	EHR, PT	Both	No	Yes	N/A	All
	**Secondary outcomes**							
		Appraisal of PCa symptoms: 4-item bother questionnaire [24,25]	SP	Both	Yes	Yes	.74-.9 [24,25]	All
		Self-efficacy in symptom management: 9-item cancer self-efficacy scale [43]	PT, SP	Both	Yes	Yes	.91-.96 [24]	All
		Social support: PROMIS SF V2.0 informational, instrumental, and emotional support scales [44]	PT, SP	Both	Yes	Yes	.74-.86 [44]	PA; CR
		Health behaviors: physical activity and nutrition in health promoting lifestyle profile II. [45-48]	PT, SP	Both	Yes	No	.75-.92 [47,48]	HB; CR
	**Antecedents (control variables): participant characteristics**							
		Demographic characteristics: age, race/ethnicity *,* income, education, and etc	PT, SP	Both	Yes	No	N/A	N/A
		Type of PCa treatment: SCP record	PT	Both	Yes	No	N/A	N/A
		Comorbidities: 13-item Charlson comorbidity index—brief [49,50]	PT, SP	Both	Yes	Yes	.73-.88 [50]	N/A
		General symptoms: 21-item Risk of Distress General Symptom Scale [51]	PT, SP	Both	Yes	Yes	.76-.84 [51]	GS
		PCa symptoms: expanded prostate cancer index composite 26 [24,52]	PT	Both	Yes	Yes	.74-.90 [53]	PCa

^a^ESCP: enhanced survivorship care plan; NCI: National Cancer Institute; PERC: prostate cancer education and resources for couples; SCP: survivorship care plan; PCa: prostate cancer related symptoms; PROMIS: Patient Reported Outcome Measures.

^b^AD: administrative data and field notes; PT: patient; SP: spouse/partner; EHR: electronic health record

^c^The elements in PERC that will impact the outcomes. PA: postsession assignment; CR: online chat room; HB: healthy behaviors (healthy eating and physical activity); GS: general symptoms of pain, fatigue, sleep disturbance, emotional distress.

^d^Both: participants in SCP only and ESCP groups.

^e^N/A: not applicable.

^f^All: all elements in PERC (mentioned above).

For quantitative data, we will obtain participants’ program satisfaction and perceived ease of use using the usability scale [39,40] and automatically recorded Web activities of the study website (access to the NCI or use of PERC). Table 1 displays the variables, measurements, and data collection information for the study.

To collect qualitative data, we will review research activity logs, field notes, participant screening data, and participant self-reported program use, as well as conduct a qualitative postintervention exit interview after study completion. In preparation for the postintervention exit interview, we will ask all participants at the T2 survey whether they are willing to talk via telephone about their experiences of using SCPs or ESCPs. Research staff will select 20 patient-partner dyads for interviews using purposeful sampling to ensure inclusion of at least one patient from each of the following groups: having/not having Internet access, having an education level of less than high school versus higher than high school, living in rural versus urban residential locations, and being African American versus white. We anticipate that these characteristics influence people’s perceptions and use of SCPs or ESCPs. Guided by a set of open-ended questions and probes, patients and partners will be interviewed together (with the telephone speaker on) and then separately (when the interviewee is alone and feels comfortable to speak freely) to learn about their shared and discrepant perceptions about the SCP or ESCP use. All interviews, conducted in a closed room, will be audio-recorded and transcribed for qualitative analysis. Research staff will also collect data about the number of visits to the genitourinary and radiation oncology clinics, patients’ primary care provider, and other providers including emergency room visits and hospitalizations.

For our secondary objective, we will evaluate participant outcomes at T1 and T2 to test the potential magnitude of benefits of the ESCP. We will obtain participant responses to structured questionnaires via telephone at T1 and T2. These Likert scales have been developed by research experts and tested for validity and reliability in previous projects [24,25,41-48]. They also demonstrated good psychometric properties in our prior studies (see Table 1 for measurements and their psychometric properties). Research staff (excluding the interventionist who knows the treatment allocation) will complete the telephone surveys where patients and their partners will be interviewed separately. Finally, we will also collect patient participant medical record data on the number of postprostate cancer treatment visits to oncologic services, their primary care providers, and other providers (including emergency room visits and hospitalizations).

#### Sample Size

There will be 25 couples each in the SCP and ESCP groups. Conservatively, we assume that we will have complete data on 23 couples per group, which is equivalent to assuming an attrition rate of 8%. Our attrition rate is based on a previous pilot study testing the feasibility of PERC in a population of patients with newly diagnosed prostate cancer and their partners recruited from the UNC Medical Center Genitourinary clinic. Unless otherwise specified, all tests will be one-sided at a .05 significance level.

### Statistical Analyses

#### Primary Objectives

For our primary objective, we base our calculations on the percentage of reviewed PERC sessions that are consistent with the symptoms patients reported, where we consider the study feasible if 80% of the reviewed PERC sessions are consistent. On the basis of our assumed sample size, we can estimate the percentage of reviewed PERC sessions that are consistent with reported symptoms with a margin of error of 16% with 95% CI. We will first use a quantitative and qualitative mixed method to analyze the data [54]. We will examine research activity logs and field notes to compute secondary feasibility measures, including enrollment, recruitment, and retention rates that will be reported by the group and by time point, along with 95% CI. Descriptive statistics (including percentages or means, standard deviations) and their corresponding 95% CIs will also be computed for participant characteristics, self-reported use of programs, and the usability scale for couples in both groups.

Interview data will be coded in atlas.ti (ATLAS.ti for Windows. Berlin: Scientific Software Development). Version 7.5.16, 1993-2019) by the investigators and the research assistants using template analysis [55]. Template analysis combines content analysis with grounded theory, applying a priori codes and allowing additional themes to emerge as analysis proceeds [55]. Members of the research team will have discussions to reconcile coding discrepancies. The responses will be analyzed based on participants’ experiences using SCP versus ESCP, to help identify the barriers and facilitators that are unique to the ESCP users. These findings will help improve the use of SCPs and ESCPs.

#### Secondary Objectives

Descriptive statistics will be calculated for secondary outcomes: QOL; appraisal of prostate cancer symptoms; self-efficacy in symptom management, social support, and health behaviors; number of patient visits to posttreatment care services; and Charlson comorbidity index, expanded prostate cancer index composite (EPIC), and General Symptom Subscale scores in the Risk of Distress Scale, for participants and partners, by time point and by group. All analyses will be conducted using an intention-to-treat approach, in which all randomized participants will be analyzed according to their assigned group, regardless of the extent of intervention received. We will use a stratified two-sample *t* test with an effect size measured by Cohen d [56] to estimate power in testing our hypotheses of greater improvement in QOL, appraisal of prostate cancer symptoms, self-efficacy in symptom management, social support, and health behaviors from T1 to T2. We will conduct complete case analysis. After accounting for about 8% attrition, an effective sample size of 23 couples per group yields 80% power to detect a moderate/large effect size of 0.74. In testing our hypothesis that SCP and ESCP patients differ in the number of visits to posttreatment care services at T2, we estimate power using a Poisson regression model. Assuming SCP patients have 6 posttreatment care visits on average at T2, a sample size of 23 patients per group yields 85% power to detect an increase or decrease of 33.3% in the average number of posttreatment care visits for patients in the ESCP group (ie, 4 or 8 posttreatment care visits at T2).

To assess the effect of group on the outcome measures for QOL, self-efficacy in symptom management, social support, and health behaviors, while accounting for the fact that couples have correlated measurements, we will fit a linear mixed model to the change score (ie, change from T1 to T2) for each outcome measure, where we will include the following as fixed effects: group membership (SCP, ESCP), couple member (patient, partner), type of prostate cancer treatment, the outcome’s measure at T1, age, income, the Charlson comorbidity index score, and the differences in the Charlson index, EPIC, and general symptom scale scores between T1 and T2. To account for the correlation between each patient and partner in each couple, we will also include couple membership as a random effect. For appraisal of symptoms, which was only measured in partners, we will fit an analysis of covariance model to its change score, including the same fixed effects as those used for QOL, self-efficacy in symptom management, social support, and health behaviors. We will fit a Poisson regression model to the number of patient visits to posttreatment care services at T2, where we will include group membership as a predictor while controlling for the following effects: treatment type, age, income, the Charlson comorbidity index, EPIC, and general symptom scale score at T2.

As this is an exploratory proof-of-concept study, rather than a confirmatory study, we will not adjust for multiplicity when computing the CIs for these feasibility measures or conduct comparisons. Unless otherwise specified, all tests will be one-sided at a .05 significance level. All analyses will be conducted using SAS 9.4 (SAS Institute Inc., Cary, NC, USA).

## Results

This project will run for 2 years and will be completed by the end of 2018. We have obtained approval from the Institutional Review Board at the two comprehensive cancer centers in the southeast United States. We have hired and trained research staff, including the project coordinator and the interventionist; set up the database using RedCap (Research Electronic Data Caputure, 2009); developed and refined protocols for all research activities; and updated the PERC website content and functionality.

We have also met with the clinical champions at the genitourinary clinics at both institutions and refined the recruitment and SCP implementation process. One of the genitourinary clinics is encountering a major nursing staff turnover, causing omission of SCP implementation to most patients at the clinic. Although the clinic is hiring new nursing staff, an oncology nurse practitioner student who is also a nurse navigator at the cancer hospital will work closely with the nurse practitioners, physician assistant, and physicians to help generate and deliver the SCPs with the embedded study website to eligible patients.

We started recruitment at the end of June 2017. As of the end of September 2017, we have consented 22 patient-partner dyads and completed the baseline survey among 16 couples. These couples have been randomly assigned to either the standardized SCP (with the NCI prostate cancer website) or the ESCP group and received the standardized SCPs. After receiving the SCPs, these couples will start their online programs. We will monitor and facilitate PERC intervention use and recruit participants for the next 6 months or until we successfully enroll 50 eligible patient-partner couples.

## Discussion

### Principal Findings

To our knowledge, this is the first study to integrate a tailored mHealth symptom self-management program with the SCP via EHR to enhance survivorship care planning and patient and family self-management. This study examines a new model of care that addresses the discord between the mandates and recommendations for survivorship care planning and scientific evidence of SCP effectiveness during care transition from end of treatment to self-management at home. We use SCPs as a vehicle to deliver mHealth programs (such as PERC) that provide a one-stop, comprehensive information and skills training mHealth intervention, as well as a place to receive social support from multiple sources (dyadic, peer, and professional) to help patients and their partners manage prostate cancer, general symptoms, and promote health behaviors. Patients and partners can conveniently access the ESCP and the mHealth program based on their preferences and needs, regardless of time and location. This study is responsive to national priorities aimed to strengthen survivorship care planning, including calls from the IOM, ACS, and Cancer Moonshot for programs that address treatment-related effects and help cancer survivors and their families to maintain QOL.

This study also addresses the great unmet supportive care needs for managing effects of cancer and its treatment for patients and families [26-29]. The substantial travel, time, and expense required to participate in in-person, multi-session, couple-focused supportive care programs limit the accessibility of these programs for patients and partners. Scalable interventions are needed to address the gap in survivorship care planning. The use of SCPs, which is increasingly required components of survivorship care planning, to deliver the tailored mHealth PERC program may facilitate referral and uptake of evidence-based mHealth programs that can reach a larger number of patients and partners at a low cost.

The innovative program has the potential to be used across diverse types of settings to address an important and frequently occurring public health problem in the US health care system as more than 220,000 men each year get diagnosed with prostate cancer. After treatment, many prostate cancer survivors experience significant physical, functional, and emotional disturbances. A scalable low-cost intervention that is widely available through routine care, EHR, and the Web, such as the ESCP, adds a significant improvement over the currently existing options.

Finally, this study innovatively involves family caregivers in posttreatment oncologic care. Family caregivers, especially partners, are often negatively affected by cancer diagnosis and treatment and sometimes have worse QOL than patients with prostate cancer [25]. Partners are a major source of support and part of the care unit [57,58]. Patients and partners often have different perceptions about prostate cancer-related issues and difficulty discussing them [59,60]. Couples lack access to survivorship care programs because of suboptimal services and referrals [61], and the small number of programs available [28] (especially those that are couple-focused [62-68]). In this study, integration of a couple-focused tailored mHealth intervention into standard SCPs is an innovative approach to deliver survivorship care information to both cancer patients and their family caregivers, and to provide family- and patient-centered care.

### Limitations

The following limitations of this study warrant discussion. First, this study is conducted in two comprehensive cancer centers, and thus, findings may have limited generalizability to settings that do not have rich resources and support (such as community cancer centers). Recognizing this inherent limitation of pilot/feasibility studies at this stage, this formative work will inform our planned study in a broader range of clinical settings to test the efficacy of the ESCP in the near future. Anecdotally smaller cancer centers and hospitals often devote specific personnel for SCP implementation to be compliant with the mandate of Commission on Cancer, which would facilitate the ESCP delivery. The additional mHealth programs such as PERC may also have more significant effects for patients receiving care at local community hospitals and cancer centers that lack personnel and resources for posttreatment care and educational resources for patients and their families.

Second, some patients and their partners may not have access to the Internet and/or a computer. We will provide cellular-ready touchscreen tablets with a 1-year 3G data plan to these couples to ensure equal access to the mHealth program.

Next, some patients and their partners may have low computer/Internet literacy, which may reduce their enthusiasm in participating in this study. To address this concern, our research staff will bring an iPad when recruiting potential participants at the clinics and show them that we designed our study website so that it requires minimum skills to navigate. Participants in our pilot studies rated our program as very simple and intuitive to use. We have developed step-by-step instructions on how to use the Internet and the study website and will provide participants these instructions by mail, email, or phone. We have also posted an instructional video on the homepage. We will provide technical support to help troubleshoot operational problems, although, in the previous testing, no pilot participants used the support.

Because PERC is a couple’s intervention that is tailored to the needs of intimate couples, we will exclude nonpartnered patients because they face different challenges than patients with partners [69]. Finally, at this time, our intervention is only available to English-speaking patients and their partners.

### Comparison With Prior Work

Compared with the standardized SCPs, ESCPs will take patients’ and partners’ specific needs into consideration and provide more detailed content that is tailored to their preferences. Implementing ESCPs will change the status quo of patients receiving relatively generic SCPs. Compared with the standardized SCPs with generic information, the ESCP will provide an empowering mHealth program (PERC) that allows patients and partners to assess their own symptoms and care needs, as well as provide resources to address their needs as they transition to posttreatment self-care at home. Compared with traditional face-to-face or telephone consultations that provide posttreatment care and education, the ESCP in this study will empower patients and their families to self-manage their symptoms and promote healthy behaviors, and thus, enable health care providers to focus their limited resources on patients who experience the most severe issues and symptoms. Survivorship care plans enhanced by a mHealth or Web-based program can also help consistently deliver posttreatment supportive care services to a larger number of patients and their families, at a low cost.

### Conclusions

The study will explore a new model of care that enhances survivorship care planning. Innovative integration of PERC with SCPs (ESCPs) will provide a tool that helps patients and partners to tailor their posttreatment symptom self-management programs based on their needs and preferences. Findings from this study will help design a definitive randomized clinical trial to test the efficacy of ESCP, a potentially scalable program.

## Abbreviations

ACS: American Cancer Society
AD: administrative data and field notes
CR: online chat room
EHR: electronic health record
EPIC: expanded prostate cancer index composite
ESCP: enhanced survivorship care plan
GS: general symptoms of pain
HB: healthy behaviors
IOM: Institute of Medicine
NCI: National Cancer Institute
PA: postsession assignment
PCa: prostate cancer related symptoms
PERC: prostate cancer education and resources for couples program
PT: patient
QOL: quality of life
SCP: survivorship care plan
SP: spouse/partner
